# Influence of volatile anaesthetics on haematology and clinical chemistry in ferrets

**DOI:** 10.1186/s12917-024-04407-y

**Published:** 2024-12-04

**Authors:** Marie-Luise Schröder, Aline Reitmeier

**Affiliations:** Department for Laboratory Animal Science, University Medical Center Hamburg-Eppendorf, Martinistrasse 52, 20246 Hamburg, Germany

**Keywords:** Isoflurane, Sevoflurane, Ferret, Anaemia, Reticulocytes

## Abstract

**Background:**

During our years of working with ferrets in our laboratory animal facility, we observed that in several healthy young female ferrets, signs of anaemia could be detected during the entry examination although none of the ferrets expressed any clinical symptoms at the time of blood withdrawal. We aimed to compare the influence of inhalation anaesthesia with isoflurane and sevoflurane to restrained, awake ferrets on several blood parameters. After arrival at our facility all ferrets received a hormone chip to subdue oestrus. Routine blood withdrawal was conducted followed by routine blood work including haematology, clinical chemistry and electrolytes. Since the size of the cannula for implementation of the hormone chip is relatively large and the insertion probably quite painful the procedure was always performed during a short inhalation anaesthesia with isoflurane or sevoflurane. In ferrets showing anaemia (haemetocrit below 0.37 l/L, haemoglobin below 11 g/dL), we performed a control blood work (venous blood) the following week. In order to rule out an effect of inhalation anaesthesia on the laboratory results, the blood withdrawal was performed in restraint and awake ferrets without anaesthesia. The study was performed as randomized controlled crossover design.

**Results:**

Thirty ferrets were enrolled, and divided in three groups. Comparison of the three methods (isoflurane, sevoflurane or restraint and awake without anaesthesia) of blood withdrawal showed statistically significant (*p* < 0.05) differences in most haematological parameters (e.g. red blood cell count, haematocrit), clinical chemistry parameters (e.g. total protein, albumin, alkaline phosphatase) and electrolytes (e.g. sodium, chloride and potassium). Restraint ferrets without anaesthesia showed no signs of anaemia. In anaesthetized ferrets, reticulocytes were about four to six times lower compared to ferrets anaesthetized with isoflurane or sevoflurane.

**Conclusions:**

The results of the study suggest that inhalation anesthesia has a significant effect on hematological and biochemical parameters in ferrets. In particular, the detection of anemia in an animal undergoing inhalation anesthesia needs to be contextualized in a clinical setting and in research context.

**Supplementary Information:**

The online version contains supplementary material available at 10.1186/s12917-024-04407-y.

## Background

In laboratory animal research, only few institutes perform studies using ferrets. Also in veterinary practice, ferrets are rare patients. Therefore, handling can be quite challenging without experience in appropriate handling methods [1]. Above all even experienced examiners sometimes struggle with the size of the animal or the availability of the vessel. After successfully puncturing the vessel, blood clotting due to slow flow rate is a frequent problem. There are different methods described for blood withdrawal: Commonly used sites include the lateral saphenous vein [2, 3] or the cephalic vein [4] while more challenging sites include the jugular vein or the cranial vena cava [5]. To minimize the stress for the ferret and the examiner likewise inhalation anaesthesia is frequently applied using a face mask or an induction chamber [6]. As anaesthetic agent, isoflurane is still the most frequently used volatile anaesthetic especially in small animal clinics due to practical reasons (costs, license) [7, 8]. Sevoflurane is mainly used within research facilities although the overall usage increases due to its lower environmental impact and superior cardiovascular properties compared to isoflurane [9, 10].

Studies analysing the influence of anaesthetics on haematological parameters in ferrets are rare [4, 11, 12]. The majority of these studies date back several years and only contain a small number of animals. In ferrets two studies dated to 1994 and 1997 describe changes in haematologic parameters in ferrets [11, 12]. Marini et al. [12] analysed the blood withdrawal in six female ferrets anaesthetized with isoflurane anaesthesia. A rapid decrease in haematocrit, red blood cell count (RBC), haemoglobin concentration occurred 15 minutes after induction of anaesthesia and partially recovered after 45 min of anaesthesia. In another study [11] splenic sequestration of red blood cells was determined as the cause of the decrease in haematological parameters. Neither the influence on reticulocytes nor the influence of sevoflurane as anaesthetic agent were investigated in these studies.

A comparison of the blood parameters with a focus on haematology including reticulocytes, clinical chemistry and electrolytes in ferrets anaesthetized either with isoflurane or sevoflurane to awake and restrained ferrets was conducted for the first time. We hypothesized a different influence of the volatile anaesthetics isoflurane and sevoflurane on the haematobiochemical parameters of ferrets. This impact should be taken into consideration when interpreting the results of haematobiochemical tests in anaesthetized subjects. The results can be an explanation to interpret blood cell count, clinical chemistry parameters and electrolytes under the respective condition – restraint and without anaesthesia versus inhalation anaesthesia.

## Methods

### Animals

Thirty clinically healthy female ferrets purchased from a commercial breeder (Euroferret, Denmark) were used. Ferrets were kept in group husbandry in floor husbandry with no special hygienic requirements and a quarterly hygienic monitoring of endo- and ectoparasites according to the Federation of European Laboratory Animal Science Associations (FELASA) recommendations [13]; a 10 / 14 h light / dark cycle, a temperature of 20 ± 2 °C and relative humidity between 50 and 60 % [13]. Animals were monitored on a daily basis, provided with different enrichment tools like thick ropes for climbing, paper boxes, bags or tunnels, every two weeks and were supplied with fresh bedding material (Hobelspäne Peer-Span, Germany) weekly; toilets (Savic cat toilet Iriz Zooplus, Germany) were cleaned and refilled (Tigerino Premium sensitive, England) at least twice per day. Food as a mixed balance of dry (Totally Ferret, Germany) and wet food (Greenwoods, Germany) and water were provided ad libitum. A period of two weeks allowed the animals to acclimate to new environmental conditions, personnel and handling. Ten ferrets each were randomly assigned to one group: blood withdrawal without anaesthesia, blood withdrawal with isoflurane anaesthesia or sevoflurane anaesthesia. Blood withdrawal (full blood cell count, clinical chemistry, electrolytes) was performed as part of the general clinical examination after arrival of new ferrets. Part of the first examination is the implementation of a hormone chip to subdue oestrus (Suprelorin^®^ Chip 4,7 mg, Virbac Tierarzneimittel GmbH). Ferrets were not fasted before the procedure. Ferrets with signs of clinical illness (e.g. dyspnoea, low body condition score with prominent bone structures, arrhythmia) in the first clinical examination, were excluded from the study. The responsible veterinarian checked the animals at least once daily for three days after blood withdrawal.

The blood withdrawal was executed within the scope of a brain function research study approved by the ethics review committee, the national authority for animal protection. It conforms to the Guide for the Care and Use of Laboratory Animals [14], eighth edition, the ARRIVE Guidelines [15] and was performed in accordance with the European Directive 2010/63 EU [14].

### Inhalation anaesthesia

**Fig. 1:**
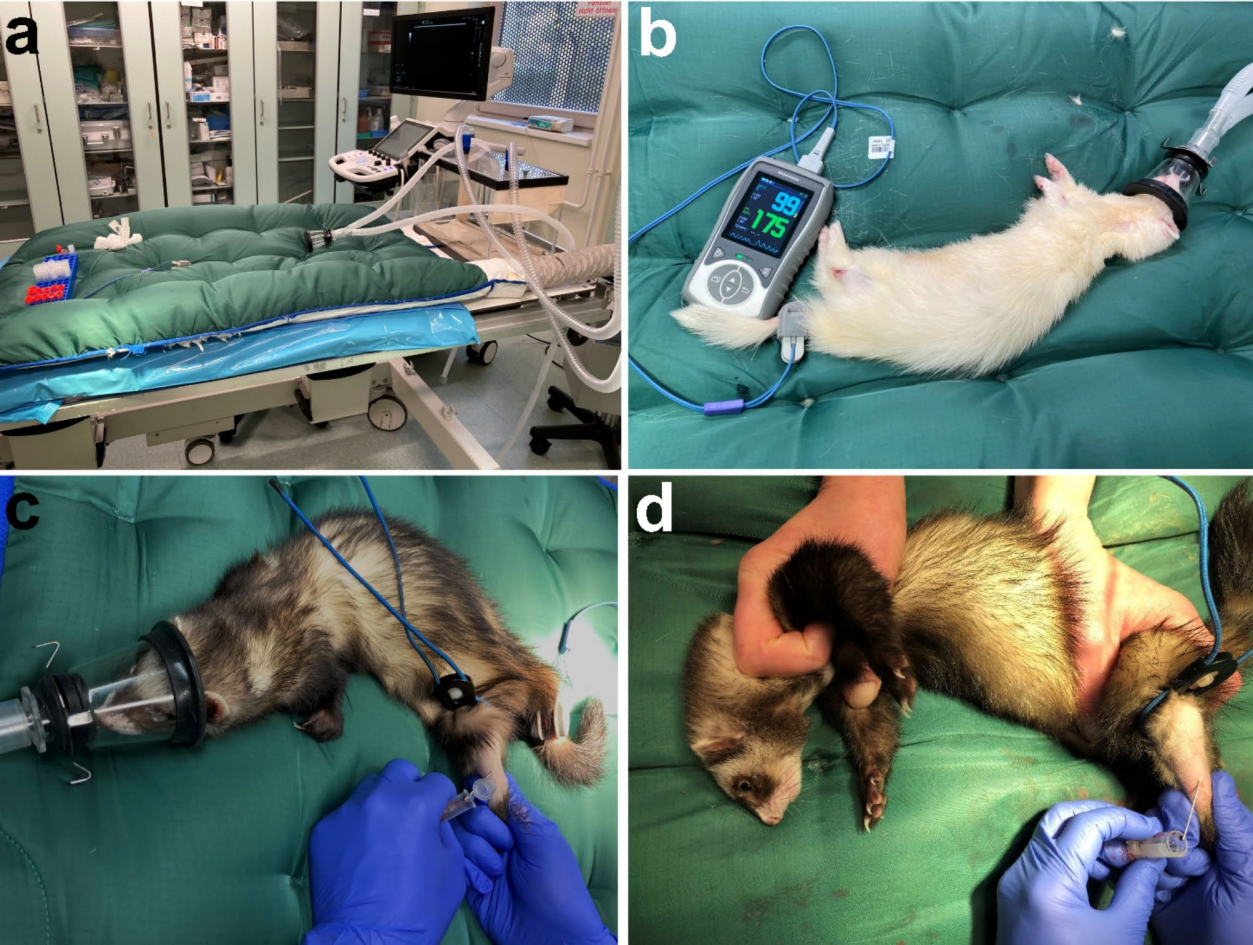
Blood collection methods in ferrets. (**a**) The set-up for the blood collection in anaesthetized ferrets, including the heating mat and all supplements necessary for the blood collection as well as the induction chamber as a transparent box on the right side of the picture. (**b**) Pulse rate and oxygen saturation monitoring using a pulse oximeter on the tail of an anaesthetized ferret, receiving anaesthetic gas through a face mask. (**c**) Blood collection from the vena saphena lateralis in an anaesthetized ferret. (**d**) Blood collection from the vena saphena lateralis in a restrained, awake ferret

For the initiation of the anaesthesia, a self-made induction chamber was used (length 40 cm, width 20 cm and height 27 cm) and preflooded with 5 % isoflurane or 8 % of sevoflurane and an oxygen flow of 4 l/min for approx. five minutes (Fig. 1a). After reaching the sufficient anaesthesia depth (constant breathing rate of 30 /min; no movement when touching; slack posture) in the induction chamber, the anaesthesia was maintained using a face mask and 3% of isoflurane or 3–4 % of sevoflurane and 0.6–1 l/min oxygen (Fig. 1b and c). For blood withdrawal, ferrets received eye ointment (Bayer, Germany) and were placed on a heating mattress (Moeck & Moeck GmbH, Germany). Heart rate and oxygen saturation were monitored via pulse oximeter (Eickemeyer KG, Germany).

### Restraining the ferret

Ferrets were restrained by either holding it by the scruff of the neck or placing one hand around the mandible and one at the back of the ferret (Fig. 1d) [1, 5, 16]. Blood withdrawal begun as soon as the ferrets started to relax. If a ferret did not relax and subordinate, blood withdrawal was not performed to avoid potential injures to the animal or human.

### Blood withdrawal

On the day of the procedure, they were randomly assorted in two standard cat carriers and afterwards transferred to the laboratory, where the ferrets had a minimum of 30 minutes to adapt before the blood withdrawal. Each method was performed exactly the same way and was always conducted by the same two veterinarians. In case puncture of the hind limb vena saphena lateralis was not successful, the vena chephalica antebrachii was punctured. Before puncturing, the region of entry was shaved and disinfected (Cutasept^®^, Hartmann AG, Germany). The conus of a 20 G cannula (Sarstedt AG & CO KG, Germany) was removed to avoid blood clotting in the conus (Fig. 1c, d) due to the inability to withdrawal blood using pressure. 700 µl blood were collected for EDTA analysis (Sarstedt AG & CO KG, Germany) and 500 µl for the analysis of clinical chemistry and electrolytes (Catalyst Lithium Heparin Whole Blood Separator, Idexx GmbH, Germany). The total collected blood volume remained below 10 % of the total blood volume recommended by the Society of Laboratory Animal Science (GV-SOLAS) [17].

### Blood analysis

EDTA blood was analyzed using the Procyte Dx Haematology Analyzer (Idexx GmbH, Germany) for the following parameters: erythrocytes, haematocrit, haemoglobin, MCV (mean corpuscular volume), MCHC (mean corpuscular haemoglobin concentration), reticulocytes, leukocytes, lymphocytes, monocytes, thrombocytes, segmented neutrophil granulocytes, eosinophilic and basophilic granulocytes. Lithium-heparin plasma (generated using the Lithium Heparin Whole Blood Separator by Idexx GmbH, Germany) was analysed using the Catalyst One Chemistry Analyzer (Idexx GmbH, Germany) and a comprehensive chemical profile (CHEM 17 CLIP, Idexx GmbH, Germany) including electrolytes (Lyte 4 CLIP, Idexx GmbH, Germany) was performed. The parameters total protein, albumin, globulin, ALT  (alanine aminotransferase), ALP (alkaline phosphatase), GGT (gamma glutamyl transferase), total bilirubin, urea nitrogen, creatinine, cholesterol, α-amylase, lipase, glucose as well as chloride, sodium, phosphorus, calcium and potassium were measured. All reference parameters were provided by IDEXX, except for GGT, α-amylase, lipase, sodium, chloride and potassium, which were transferred from Hein et al. 2012 [3].

### Statistical methods

Ferrets were randomly assigned to three group (blood withdrawal without anaesthesia, blood withdrawal with isoflurane anaesthesia or with sevoflurane anaesthesia) of ten ferrets according to their age. Normally distributed variables are reported as mean ± standard deviation, not normally distributed values are displayed as median (minimum - maximum). Each parameter was analysed independently in a group comparison within the three groups (restrain, isoflurane, sevolfurane). Data were analysed for normal distribution using Shapiro Wilk test and visually assessed using Q-Q plots. Subsequently, one-way ANOVA followed by Tukey´s multiple comparisons test was used for normal distributed results and Kruskal Wallis followed by Dunn’s test were used for non-normally distributed results. A p-value ≤ 0.05 was considered statistically significant. The results are displayed as boxplots. The boxes represent the 25th to 75th percentiles, and the centerline indicates the median. The whiskers display the minimum and maximum values. Graphpad Prism 9 (Graphpad Inc., USA) was used for graphical presentation of the results and statistical analyses.

## Results

### Animals

Clinically healthy female ferrets (*n* = 10 per group, 756 ± 97 g body weight, 59 ± 0.8 weeks old) were used for this study. No animal had to be excluded from this study due to a clinical illness. No ferret was excluded of the study due to uncooperative behavior during restraining.

### Anaesthesia

Inhalation anaesthesia (isoflurane and sevoflurane) was successful with no complications in all ferrets. No cardiorespiratory depression (pulse rate below 200 pulses per minute, respiratory rate below 33 breaths per minute, oxygen saturation below 95 %) was noted. Animals were monitored until they were fully awake moving normally, pulse rate and breathing rate within normal range (pulse rate 200–400 per minute, respiratory rate 33–36 per minute). The general time for a ferret to reach a sufficient stage of anaesthesia was 1.24 ± 0.01 minutes for isoflurane anaesthesia and 1.29 ± 0.02 minutes for sevoflurane anaesthesia. Mean pulse rate was 250 ± 19 bpm (beats per minute) for isoflurane anaesthesia and 207 ± 28 bpm for sevoflurane anaesthesia. Oxygen saturation was always above 95 %.

### Duration of the blood withdrawal

Blood was successfully collected in 90 % of ferrets from the vena saphena lateralis without any difficulties. In the remaining 10 % of ferrets (*n* = 2 anaesthetized with isoflurane and *n* = 1 anaesthetized with sevoflurane), blood was collected from the vena cephalica antebrachii due to insufficient collected blood volumina of the vena saphena lateralis. A statistically significant difference was found in the time required to achieve blood collection of ferrets without anesthesia and anesthetized ferrets: blood was collected within 2.04 (1.50–6.83) minutes from ferrets without anaesthesia, compared to 11.86 (7.97–21.40) minutes with isoflurane (*p* < 0.0009) and 14.2 (6.83–28.33) minutes with sevoflurane (*p* < 0.0002).

Median blood collection time was 1.18 (1.00–6.15) minutes in restraint ferrets without anaesthesia, 2.29 (1.00–6.67) minutes in ferrets anaesthetized with isoflurane, and 1.55 (0.50–18.9) minutes in ferrets anaesthetized with sevoflurane. No statistical difference was found comparing restraint non-anaesthetized to anaesthetized groups (*p* > 0.05).

### Haematology

**Fig. 2:**
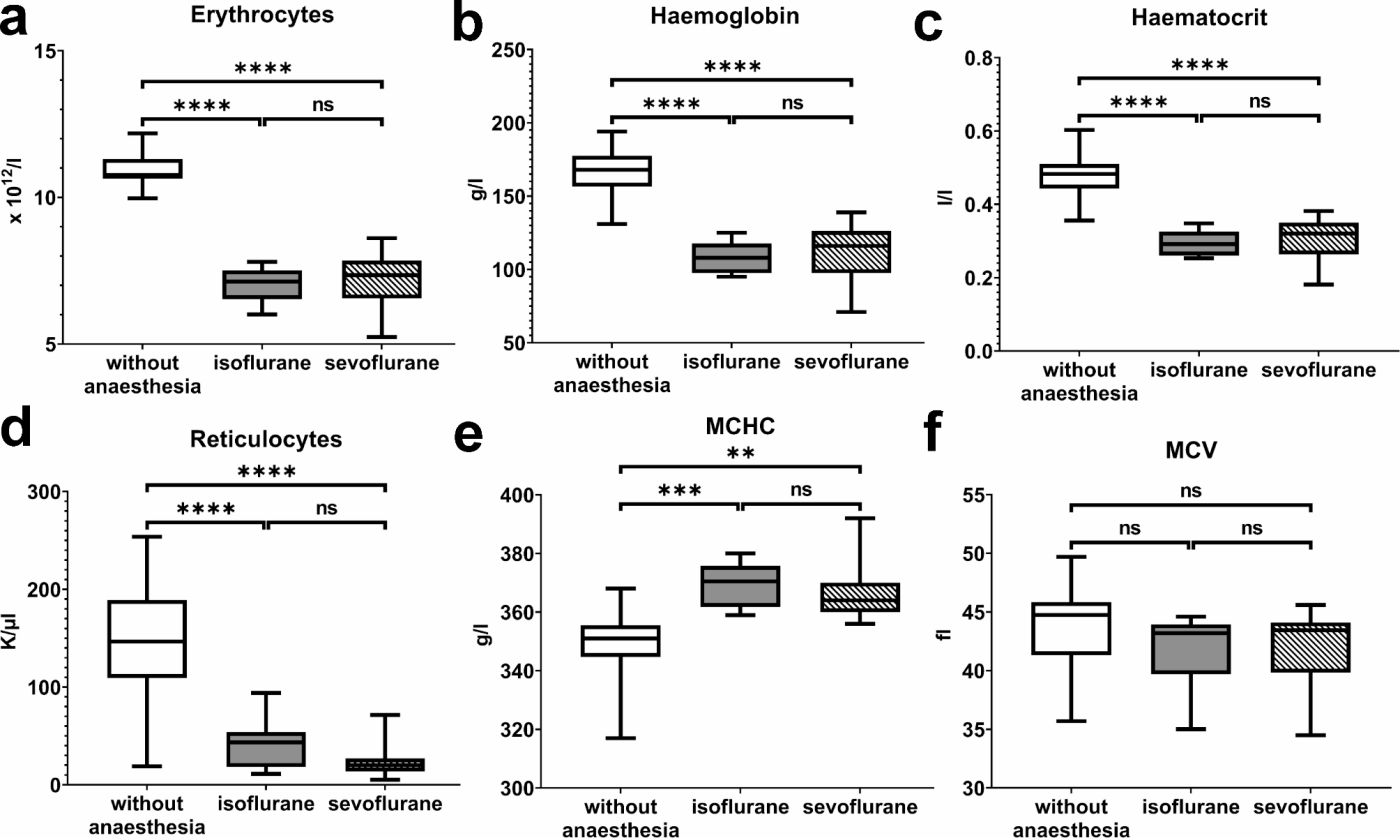
Differences of red blood cell parameters in ferret EDTA blood samples, which were obtained in ferrets without anaesthesia or after anaesthesia via isoflurane or sevoflurane: red blood cell count (**a**), haemoglobin concentration (**b**), haematocrit (**c**), reticulocytes (**d**) and MCHC - mean corpuscular haemoglobin concentration (**e**), MCV - mean corpuscular volume (**f**), ** ≤ 0.01, *** ≤ 0.001, **** ≤ 0.0001, ns – no statistically significant difference with *p* > 0.05

All hematologic parameters were measurable in all animals, except for three thrombocyte counts. Thrombocyte counts that contained clots and were excluded from the statistical analysis (*n* = 1 for isoflurane and *n* = 2 for sevoflurane). Table 1 shows the detailed results of the haematological analysis of EDTA blood collected from restraint ferrets without anaesthesia and anaesthetized ferrets using isoflurane and sevoflurane and Fig. 2 shows the differences in the hematologic parameters. Statistically significant differences were detected in the haematological parameters among groups. The RBC count, haemoglobin concentration, haematocrit and number of reticulocytes were statistically significantly lower in anaesthetized ferrets compared to restraint ferrets without anaesthesia, whereas the MCHC was statistically significantly higher in anaesthetized animals using isoflurane compared to restraint ferrets without anaesthesia. The MCV and the white blood cell count showed no statistically significant differences between the restraint ferrets without anaesthesia and the ferrets anaesthetized with isoflurane and sevoflurane.

**Table 1:** Hematological parameters in ferrets and comparison among three groups (without anesthesia, anesthetized with isoflurane, anesthetized with sevoflurane)

Parameter	Unit	Reference interval	Without anesthesia	Isoflurane	Sevoflurane	Group comparison
Erythrocytes	x 10^12^/L	6.35–11.20	10.97 ± 0.65	7.01 ± 0,56	7.22 ± 0.91	a (*p* < 0.0001), b (*p* < 0.0001)
Haemoglobine	g/L	110–170	166.60 ± 18.07	107.60 ± 9.96	93.55 ± 45.36	a (*p* < 0.0001), b (*p* < 0.0001)
Haematocrit	L/L	0.37–0.55	0.48 ± 0.07	0.29 ± 0.03	0.27 ± 0.07	a (*p* < 0.0001), b (*p* < 0.0001)
MCV	fL	45–55	44.75 (35.70–49.70)	43.20 (35.00–44.60)	43.45 (34.50–45.60)	ns (*p* > 0.05)
MCH	pg	14–18	15.04 ± 1.06	15.36 ± 1.03	15.30 ± 1.10	ns (*p* > 0.05)
MCHC	g/L	320–350	348.50 ± 12.64	369.10 ± 6.96	366.70 ± 9.56	a (*p* = 0.0005), b (*p* = 0.0018)
Reticulocytes	K/µL	0,0–60	147.67 ± 66.28	36.44 ± 19.53	23.89 ± 17.27	a (*p* < 0.0001), b (*p* < 0.0001)
Leukocytes	x 10^9^/L	2–10	7.83 ± 2.67	7.96 ± 2.85	7.26 ± 2.91	ns (*p* > 0.05)
Lymphocytes	x 10^9^/L	1–8	3.14 (2.09–8.21)	3.91 (1.47–5.50)	2.99 (1.54–7.68)	ns (*p* > 0.05)
Thrombocytes	x 1^09^/L	270–880	689.20 ± 281.27	423.50 ± 185.59	414.00 ± 221.73	ns (*p* > 0.05)
Monocytes	x 10^9^/L	0.18–0.90	0.51 (0.31–0.83)	0.05 (0.28–1.17)	0.37 (0.20–1.06)	ns (*p* > 0.05)
Segmented Neutrophil Granulocytes	x 10^9^/L	0.62–3.30	2.55 (1.22–6.33)	2.71 (1.41–6.11)	2.13 (1.15–5.00)	ns (*p* > 0.05)
Eosinophil Granulocytes	x 10^9^/L	0.10–0.60	0.16 ± 0.12	0.16 ± 0.10	0.29 ± 0.32	ns (*p* > 0.05)
Basophil Granulocytes	x 10^9^/L	0.00–0.10	0.04 (0.00–0.21)	0.02 (0.00–0.06)	0.03 (0.00–0.12)	ns (*p* > 0.05)

Normally distributed variables are reported as mean ± standard deviation, not normally distributed values are displayed as median (minimum - maximum). Statistically significant differences of group comparisons with corresponding p-values are described as follows: (ns): non statistically significant difference, (a) statistically significant difference between ferrets without anesthesia and ferrets anesthetized with isoflurane, (b) statistically significant difference between ferrets without anesthesia and ferrets anesthetized with sevoflurane (c). Abbreviations are defined as follows: MCV (mean corpuscular volume), MCH (mean corpuscular haemoglobin), MCHC (mean corpuscular haemoglobin concentration)

### Clinical chemistry

**Fig. 3:**
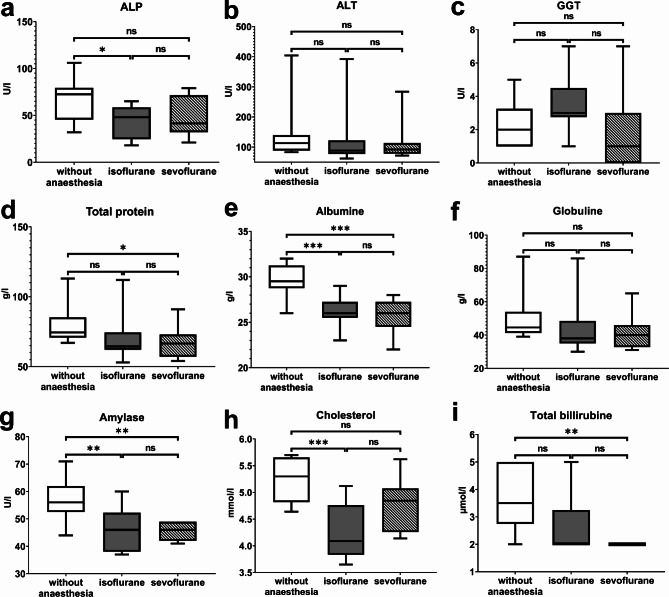
Parameters of the clinical chemical analysis of ferret plasma samples, which were obtained in ferrets without anaesthesia or after anaesthesia via isoflurane or sevoflurane: ALP - alkaline phosphatase (**a**), ALT - alanine aminotransferase (**b**), GGT - gamma glutamyl transferase (**c**), total protein (**d**), albumine (**e**), globuline (**f**), amylase (**g**), cholesterol (**h**), total bilirubine (**i**), * ≤ 0.05, ** ≤ 0.01, *** ≤ 0.001, ns – no statistically significant difference with *p* > 0.05

All clinical chemistry parameters were measurable in all animals. Table 2 shows detailed results and results of the statistical comparisons of the clinical chemical analysis of plasma collected from ferrets without anaesthesia and anaesthetized ferrets using isoflurane and sevoflurane. Figure 3 shows statistically significant differences of clinical chemistry parameters. Plasma concentrations of total protein and albumin and ALP were statistically significantly lower in blood samples of anaesthetized ferrets (isoflurane or sevoflurane) compared to serum samples of ferrets without anaesthesia. In contrast, the globulin as well as the ALT und GGT concentrations showed no statistically significant differences in restraint ferrets without anaesthesia or anaesthetized ferrets.

Total bilirubin plasma levels were statistically significantly lower in blood samples of ferrets anaesthetized with isoflurane or sevoflurane compared to restraint and awake ferrets without anaesthesia. Cholesterol levels in the plasma of ferrets without anaesthesia were statistically significant higher compared to blood obtained from ferrets under isoflurane anaesthesia. Concentrations of α-amylase were statistically significantly higher in the plasma of restraint, awake ferrets without anaesthesia compared to anaesthetized ferrets.

Plasma concentrations of urea nitrogen, creatinine, lipase and glucose showed no statistically significant differences in blood samples of restraint, awake ferrets without anaesthesia compared to anaesthetized ferrets. All plasma parameters, which showed no statistically significant differences, are displayed in the supplements (S2).

**Table 2:** Clinical chemistry parameters in ferrets and comparison among three groups (without anesthesia, anesthetized with isoflurane, anesthetized with sevoflurane)

Parameter	Unit	Reference Interval	Without Anesthesia	Isoflurane	Sevoflurane	Group Comparison
Total Protein	g/L	52–73	74.50 (67.00–113.00)	64.50 (53.00–112.00)	66.50 (54.00–91.00)	b (*p* = 0.0425)
Albumin	g/L	26–38	29.60 ± 1.74	26.10 ± 1.64	25.70 ± 1.90	a (*p* = 0.0007), b (*p* = 0.0002)
Globulin	g/L	18–31	44.50 (39.00–87.00)	38.00 (30.00–86.00)	40.00 (31.00–65.00)	ns (*p* > 0.05)
ALT	U/L	82–289	113.00 (83.00–404.00)	88.50 (62.00–392.00)	92.00 (72.00–284.00)	ns (*p* > 0.05)
ALP	U/L	9–84	67.30 ± 20.87	42.60 ± 16.55	48.00 ± 19.69	a (*p* = 0.0281)
GGT	U/L	0.2–14	2.20 ± 1.33	3.60 ± 1.69	1.80 ± 2.04	ns (*p* > 0.05)
Total Bilirubin	µmol/L	2–17	3.50 (2.00–5.00)	2.00 (2.00–5.00)	2.00 (2.00–2.00)	b (*p* = 0.0012)
Urea Nitrogen	mmol/L	3.6–16.1	7.89 ± 1.58	8.09 ± 0.97	7.72 ± 1.22	ns (*p* > 0.05)
Creatinine	µmol/L	35–80	41.70 ± 7.60	39.50 ± 14.17	37.20 ± 8.16	ns (*p* > 0.05)
Cholesterol	mmol/L	1.65–7.64	5.25 ± 0.37	4.28 ± 0.50	4.75 ± 0.44	a (*p* = 0.0002)
α-Amylase	U/L	19.4–61.9	56.80 ± 7.18	40.85 ± 14.03	45.60 ± 3.01	a (*p* = 0.0035), b (*p* = 0.0025)
Lipase	U/L	73.2-351.1	1887.50 ± 647.91	1440.20 ± 289.65	1488.30 ± 450.78	ns (*p* > 0.05)
Glucose	mmol/L	5.23–11.51	6.69 ± 0.82	6.42 ± 0.88	6.78 ± 0.70	ns (*p* > 0.05)

Normally distributed variables are reported as mean ± standard deviation (SD), not normally distributed values are displayed as median (minimum - maximum). Statistically significant differences of group comparisons with corresponding p-values are described as follows: (ns): non statistically significant difference, (a) statistically significant difference between ferrets without anesthesia and ferrets anesthetized with isoflurane, (b) statistically significant difference between ferrets without anesthesia and ferrets anesthetized with sevoflurane (c). Abbreviations are defined as follows: alanine aminotransferase (ALT), alkaline phosphatase (ALP), gamma glutamyl transferase (GGT)

### Electrolytes

**Fig. 4:**
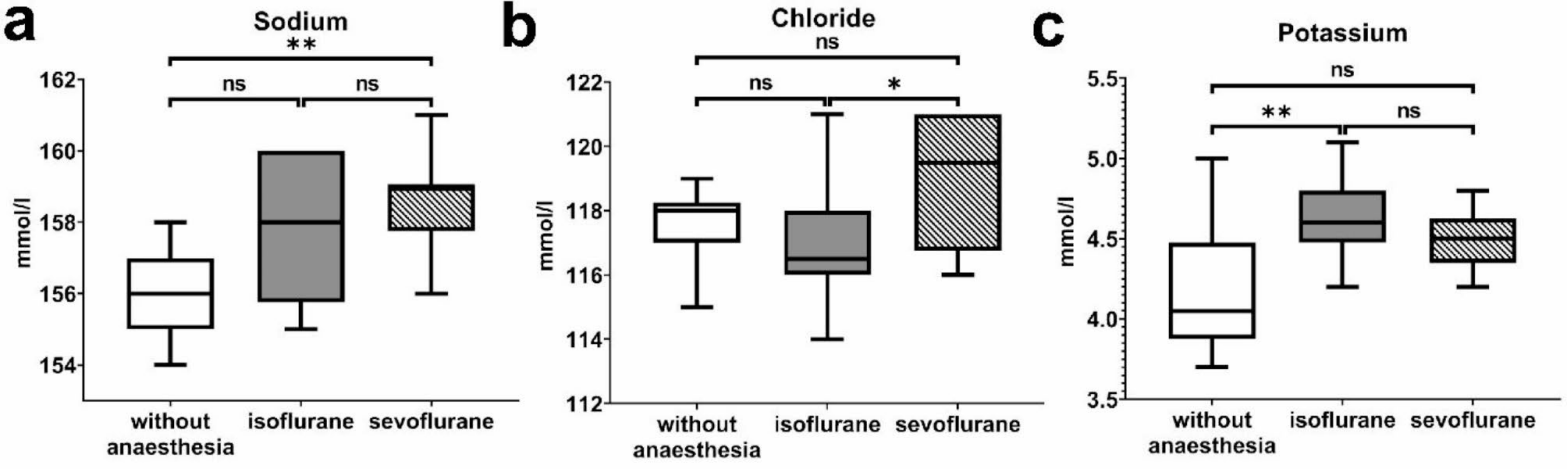
Measurement of electrolyte concentrations in ferret plasma samples obtained in ferrets without or anaesthesia after anaesthesia via isoflurane or sevoflurane: sodium (**a**), chloride (**b**), potassium (**c**), * ≤ 0.05, ** ≤ 0.01, ns – no statistically significant difference with *p* > 0.05

All electrolytes were quantifiable in all animals. The detailed results of the analysis of plasma electrolyte samples collected from ferrets without anaesthesia and anaesthetized ferrets using isoflurane and sevoflurane are displayed in Table 3. Figure 4 shows the statistically significant differences in electrolyte concentrations. Statistically significantly higher levels of plasma sodium were detected in blood samples of ferrets anaesthetized with sevoflurane compared to ferrets without anaesthesia. The plasma chloride concentration was statistically significantly higher in ferrets anaesthetized with sevoflurane compared to ferrets anaesthetized with isoflurane. Plasma levels of potassium were statistically significantly higher in ferrets anaesthetized with isoflurane compared to restraint ferrets without anaesthesia. Phosphate and calcium plasma levels showed no statistically significant differences in ferrets without anaesthesia compared to anaesthetized ferrets. Plasma levels with no statistically significant differences are displayed in the supplements (S3).

**Table 3:** Electrolytic parameters in ferrets and comparison among three groups (without anesthesia, anesthetized with isoflurane, anesthetized with sevoflurane)

Parameter	Unit	Reference interval	Without anesthesia	Isoflurane	Sevoflurane	Group comparison
Sodium	mmol/L	140.1–169.7	156.00 ± 1.18	157.70 ± 1.85	158.50 ± 1.28	b (*p* = 0.0034)
Chloride	mmol/L	108–119.9	117.60 ± 1.11	116.90 ± 1.76	118.90 ± 1.92	c (*p* = 0.0389)
Potassium	mmol/L	3.9–5.9	4.18 ± 0.4	4.63 ± 0.24	4.49 ± 0.19	a (*p* = 0.0074)
Phosphate	mmol/L	1.55–2.87	1.73 ± 0.48	1.54 ± 0.32	1.68 ± 0.38	ns (*p* > 0.05)
Calcium	mmol/L	2.00–2.95	2.24 (2.19–2.78)	2.16 (2.00–2.42)	2.16 (2.03–2.43)	ns (*p* > 0.05)

Normally distributed variables are reported as mean ± standard deviation (SD), not normally distributed values are displayed as median (minimum - maximum). Statistically significant differences of group comparisons with corresponding p-values are described as follows: (ns): non statistically significant difference, (a) statistically significant difference between ferrets without anesthesia and ferrets anesthetized with isoflurane, (b) statistically significant difference between ferrets without anesthesia and ferrets anesthetized with sevoflurane (c)

## Discussion

The aim of the current study was to compare the blood work results between awake and anaesthetized healthy ferrets and therefore the effect of aesthesia on blood parameters.

During our years of working with ferrets, clinical healthy ferrets frequently showed signs of anaemia in the haemogram (decreased erythrocytes, haematocrit and haemoglobin) during routine blood work (ProCyte Dx, Catalyst One, Idexx Veterinary Laboratories) without showing any clinical symptoms of anaemia like weakness, pallor or disorientation. The blood collection is always performed using volatile anaesthetics, which is necessary since a painful implementation of a hormone chip to subdue oestrus is part of the entry examination at arrival at our facility. The following week, signs of anaemia always disappeared in the control haemogram performed without anaesthesia. Since restraining a ferret can be quite challenging especially with not tamed, non-cooperative animals or during painful procedures [6], using inhalation anaesthesia (mainly isoflurane), is a commonly used technique in animal clinics or research facilities [8]. Adverse effects, as decreased haematological parameters were described using isoflurane anaesthesia. We detected differences in the haemogram (erythrocytes, haemoglobin, haematocrit, MCHC, reticulocytes), clinical chemical parameters (total protein, albumin, ALP, total bilirubin, cholesterol, α-amylase), and electrolyte levels (sodium, chloride, and potassium) when comparing blood samples from ferrets without anaesthesia to those from anaesthetized ferrets in our study.

Nevertheless, no studies we found compared blood parameters with isoflurane anaesthesia, sevoflurane anaesthesia and no use of inhalation anaesthesia during blood collection.

Within the scope of our study the use of inhalation agent always led to a decrease of haematocrit and haemoglobin below reference values, erythrocytes remained at the lower spectrum of the reference values, reticulocytes (absolute count) remained within reference values. In no case clinical signs of anaemia could be observed. Marini et al. [12] described a decrease in these haematological parameters 15 min after induction of anaesthesia and a partial recovery after 45 min of anaesthesia. Our study shows a decrease in these haematological parameters at an earlier time point immediately after induction of anaesthesia. Therefore, the effects are already present using short-term inhalation anaesthesia. Marini et al. [12] considered several possible causes for reductions in haematological variables during anaesthesia like pooling of blood, compartmental fluid shifts, and selective sequestration of cellular elements or hypotension with change in organ blood flow. In a follow-up study by Marini et al. [11] splenic sequestration of red blood cells was determined as the cause of the decrease in haematological parameters. The authors concluded that after phenylephrine injection vasomotor changes as well as smooth muscle contraction in the spleen led to an increase in red blood cells. Lower values of RBC, haematocrit, haemoglobin and higher levels of MCHC were detected in anesthetized ferrets. These findings could be a result of haemolysis. On the other hand, plasma blood samples showed no signs of haemolysis, e.g. a red staining of the plasma. Therefore, we conclude that the found changes are a result of the applied anaesthesia. However, an examination of blood smears would have given additional insights into the interpretation of the presented data of this study.

To our knowledge there are no data showing the influence of sevoflurane on blood parameters in ferrets. We could prove in our study that the use of sevoflurane causes similar adverse effects on haematology in comparison to isoflurane. Although the cardiovascular influence of sevoflurane is known to be less severe compared to isoflurane [18] their overall characteristics are similar [10]. Therefore, the anaesthetics could cause the effects on blood parameter changes in the samples of anaesthetized ferrets.

Reticulocytes in awake ferrets were significantly higher compared to the anaesthetized ferrets. The influence of anaesthesia on reticulocyte count was never analysed before. Common pathological causes for increased numbers of reticulocytes are haemolysis, blood loss (regenerative anaemia) or hypoxia [19], which were likely to not occur in our ferrets. Reticulocytosis in non-anaemic veterinary patients is well described and a topic of interested in cats and dogs, but was never shown in ferrets before [19–21]. There is speculation that in cats, dogs and rats the result is caused by either activation of the reticuloendothelial system, vasoconstriction of the marrow blood vessels or stimulation of the suprarenal glands, spleen, or other erythropoetin-producing organs due to stress, exercise or an underlying disease [22–24]. Recent studies report a mortality rate of up to one third in these veterinary patients and indicate that a reticulocytosis in non-anaemic veterinary patients could be stress induced but also a sign of underlying disease [20]. The question remains, if volatile anaesthetics mask reticulocytosis or if restraining led to higher reticulocyte count due to catecholamine distribution even in animals used to handling. The findings in our study support an increased awareness to monitor the reticulocyte count more carefully in context to the patient. A careful evaluation of pros and cons of either using volatile anaesthetics (isoflurane, sevoflurane) or restrain an awake ferret is advisable for diagnostics. The correct diagnosis of anaemia is not restricted to a clinical set-up, but is also important in ferrets used in research, e.g. due to blood loss during or after surgical interventions, the experimental design (drug-inducing anaemia), or due to the onset of a chronic disease in animals used for specific research questions. Accurate health evaluation of the patient is crucial since already sick veterinary patients might not tolerate an intervention affecting the red blood count. Moreover, the usage of an animal with underlying disease could change the outcomes of the study at worst and thereby reducing transferability. Whereas whenever blood withdrawal in clinical healthy ferrets is performed under isoflurane or sevoflurane anaesthesia, red blood parameters should be reviewed carefully since decreased values of haematocrit, haemoglobin and erythrocytes could potentially mislead to the conclusion of an anaemic animal or the beginning or existence of a chronic disease. The results of this study underline the importance of whether volatile anaesthetics were used or if the ferret was restrained and awake when analysing and interpreting the results.

A decrease in albumin, globulin, ALP and ALT was detected in our study in anaesthetized ferrets compared to restraint ferrets without anaesthesia. Sodium and potassium were higher in anaesthetized compared to restraint ferrets without anaesthesia, whereas the results of chloride were inconclusive. The interpretation of differences in plasma chemical parameters and electrolytes is quite challenging, because studies analysing the effect of anaesthetics on plasma chemistry and electrolytes were only conducted in mini pigs, mice or guinea pigs [25–27]. Total protein was the only parameter analysed in ferrets, which were restraint or anaesthetized with isoflurane and showed a similar decrease compared to our study [12]. Differences in the anaesthetic regimen complicate the comparison of studies since additional medications change the results of the blood examination. Moreover interspecies differences between omnivores, herbivores and carnivores and their physiological differences complicate the interpretation of the results.

Several studies provide reference intervals for ferrets which vary depending on different parameters [3, 4, 28–30]. Considering the findings of this study, reference values provided within the scope of other studies can merely be used as an orientation whilst interpreting results of the own study highlight the importance of control groups. The influence of the laboratory process, form of sampling and analysis can have a statistically significant influence on the measurement values and have to be considered as well. The results of this study underline the importance of internal standards and internal reference intervals as well as the knowledge about the creation of reference values (e.g. awake or anaesthetized ferrets) and show the importance of a careful interpretation of the results within the scope of a study or a clinical situation. It is not clear if there is a sex-based influence on the results since only female ferrets were included. In humans, an influence of hormones on the RBC was shown [30]. If we apply the results of the study in humans to ferrets, there should also be a sex-dependent influence on the RBC. To our knowledge the hormonal influence on ferrets has not yet been analysed. Therefore, further studies using male and female ferrets have to evaluate the potential limitation of the animal model in this study as reported for haematological parameters and a limited number of serum parameters in ferrets. No statistically significant age-related difference was observed in this study and another study [31], but was shown in other species [32]. We chose young adult ferrets of approximately one year of age to exclude a potential impact of age on the results because age was similar in all groups. Further studies need to investigate the influence of age with or without the effect of inhalation anaesthesia on blood parameters in ferrets.

## Conclusion

The anaesthetic agent can have a statistically significant influence on the haemogram as well as some plasma parameters and electrolytes. Especially RBC parameters, but also clinical chemical parameters and electrolytes were alleviated in blood samples of anaesthetized ferrets. Reticulocytes showed an elevation in restraint, awake ferrets without anaesthesia, which could be, similar to cats and dogs, stress-induced. The results of this study emphasise the importance of a thoughtful study design and interpretation of results in a clinical situation as well as in research studies.

## Electronic supplementary material

Below is the link to the electronic supplementary material.

Supplementary Material 1

## Data Availability

The authors declare that the data supporting the findings of this study are available within the paper and its Supplementary Information files. Raw data files in another format are available from the corresponding author upon reasonable request.
