# Supplementary material for: Influence of volatile anaesthetics on haematology and clinical chemistry in ferrets
Source: BMC Vet Res. 2024 Dec 4;20:551. doi: 10.1186/s12917-024-04407-y (PMC11616128; doi:10.1186/s12917-024-04407-y)
Supplement: Supplementary file 1 — Supplementary Material 1 [file 12917_2024_4407_MOESM1_ESM.docx]

**Supplemental material**

**
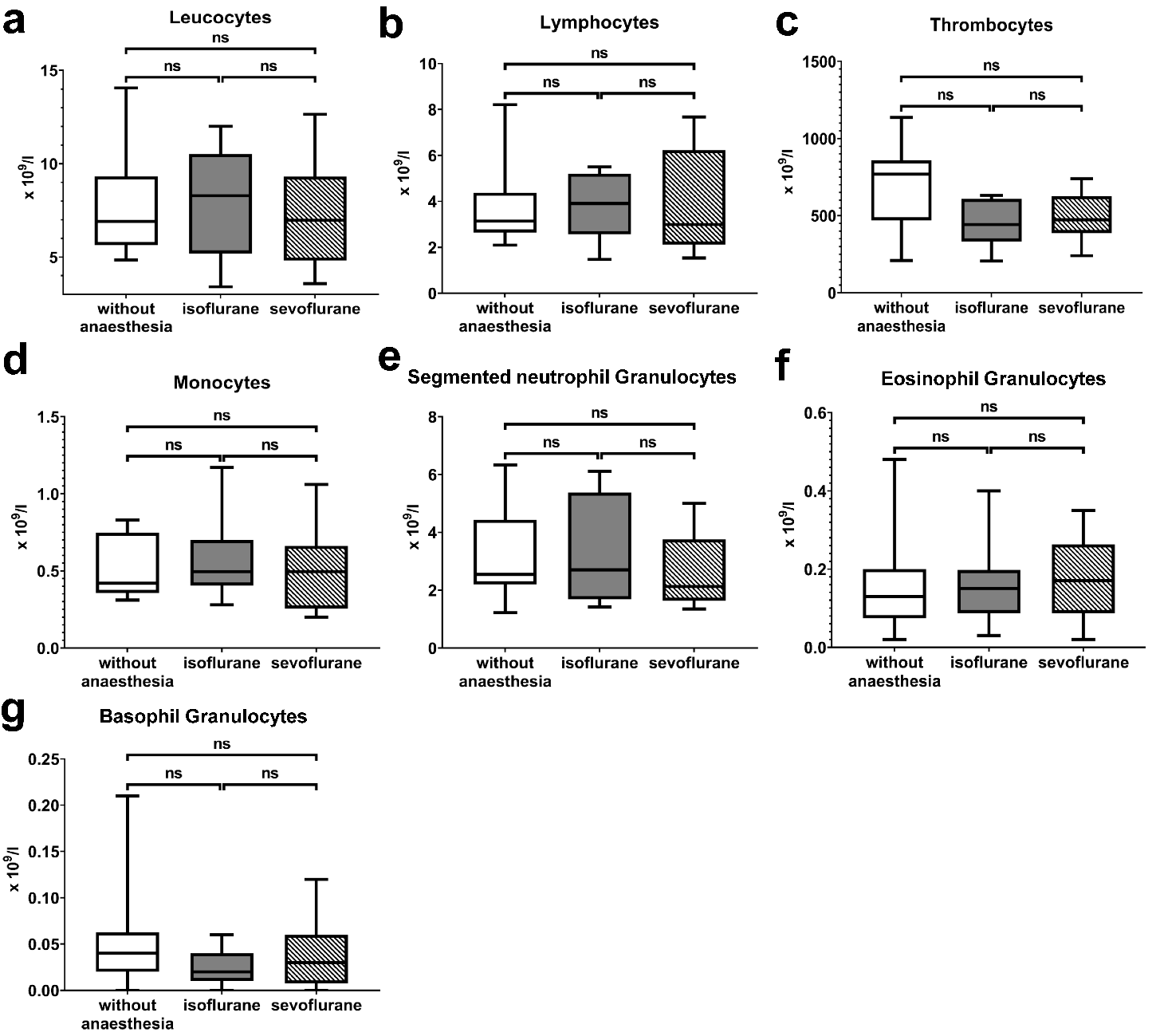
**

S1. White blood cell parameters. No significant differences could be detected in the concentration of leucocytes (a), lymphocytes (b), thrombocytes (c), monocytes (d), segmented neutrophil granulocytes (e), eosinophil granulocytes (f) and basophil granulocytes (g) in ferrets anaesthetised with isoflurane or sevoflurane compared to ferrets without anaesthesia, which were simply restraint. ns – not significant

**
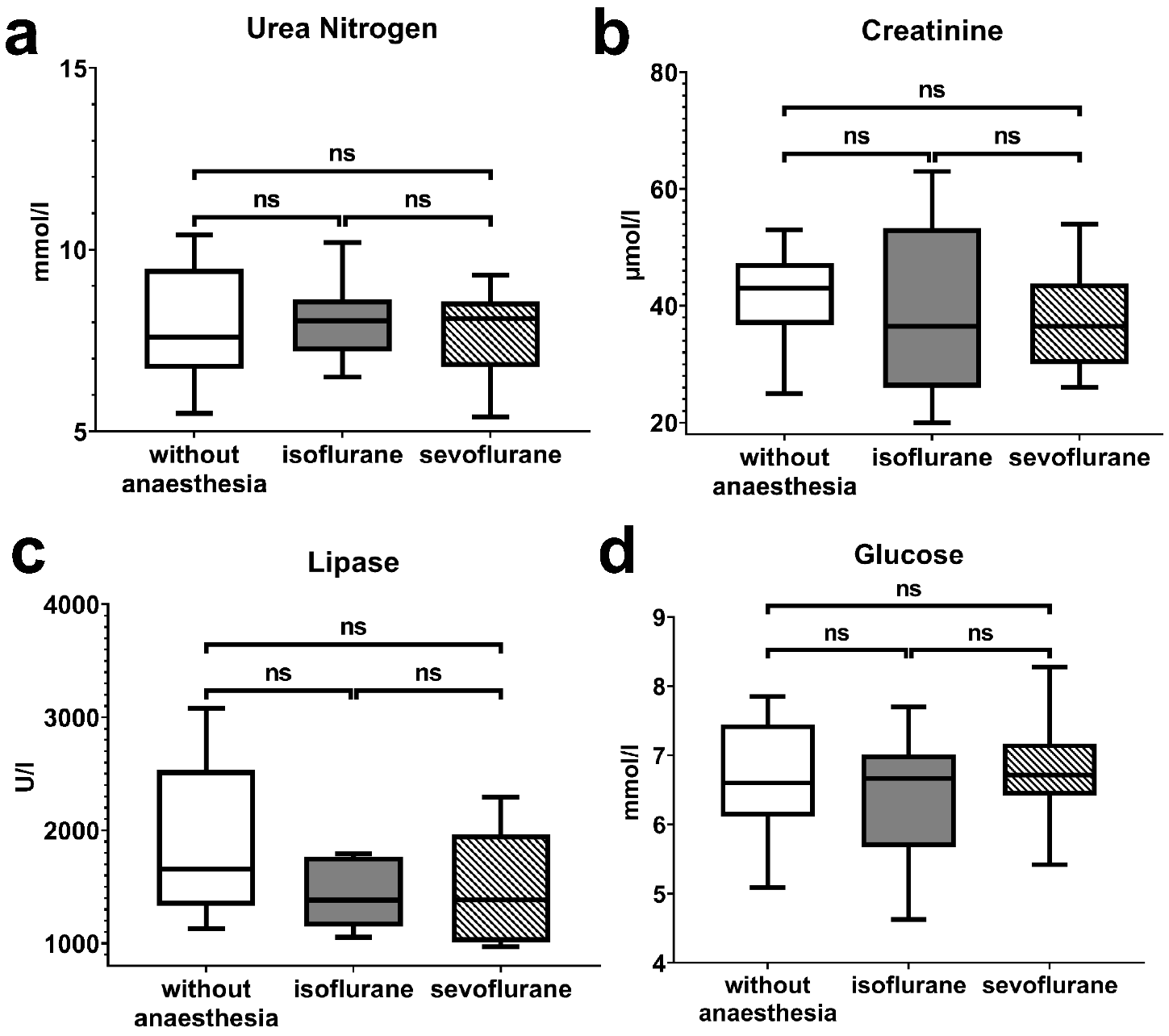
**

S2. Additional clinical chemical parameters and electrolytes. The concentration of urea nitrogen (a), creatinine (b), lipase (c) and glucose (d) showed no significant differences in ferrets anaesthetised with isoflurane or sevoflurane compared to ferrets without anaesthesia, which were simply restraint. ns – not significant

**
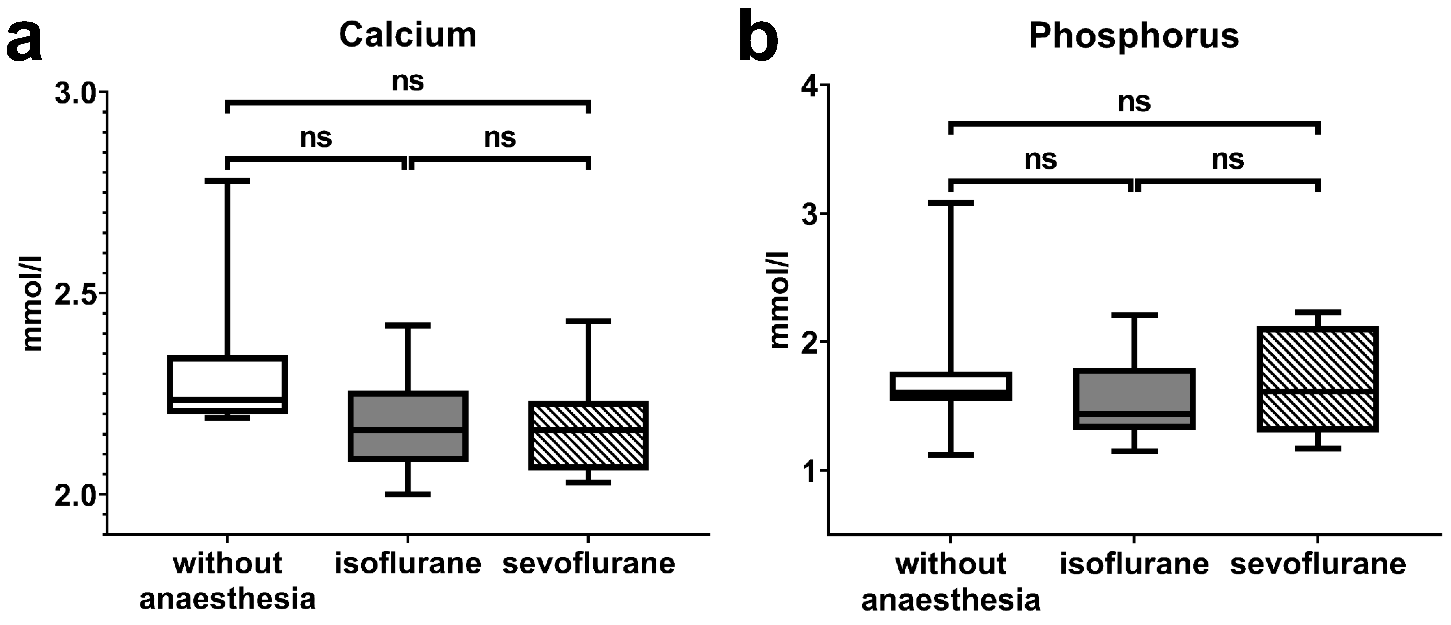
**

S3. Additional measurements of electrolytes. The concentration of calcium (a) and phosphorus (b) showed no significant differences in ferrets anaesthetised with isoflurane or sevoflurane compared to ferrets without anaesthesia, which were simply restraint. ns – not significant
